# All Our Babies Cohort Study: recruitment of a cohort to predict women at risk of preterm birth through the examination of gene expression profiles and the environment

**DOI:** 10.1186/1471-2393-10-87

**Published:** 2010-12-30

**Authors:** Sara K Gracie, Andrew W Lyon, Heather L Kehler, Craig E Pennell, Siobhan M Dolan, Deborah A McNeil, Jodi E Siever, Sheila W McDonald, Alan D Bocking, Stephen J Lye, Kathy M Hegadoren, David M Olson, Suzanne C Tough

**Affiliations:** 1 Department of Obstetrics and Gynecology, University of Alberta, Edmonton, Alberta, Canada; 2 Department of Pathology and Laboratory Medicine, University of Calgary, Calgary, Alberta, Canada; 3 Division of Clinical Pathology, Calgary Laboratory Services, Calgary, Alberta, Canada; 4 Public Health, Innovation and Decision Support, Alberta Health Services, Calgary, Alberta, Canada; 5 School of Women's and Infants' Health, University of Western Australia, Perth, Western Australia, Australia; 6 Department of Obstetrics & Gynecology and Women's Health, Albert Einstein College of Medicine/Montefiore Medical Center, New York, New York, USA; 7 Department of Paediatrics, University of Calgary, Calgary, Alberta, Canada; 8 Department of Obstetrics and Gynecology, University of Toronto, Toronto, Ontario, Canada; 9 Samuel Lunefeld Research Institute, University of Toronto, Toronto, Ontario Canada; 10 Faculty of Nursing, University of Alberta, Edmonton, Alberta, Canada; 11 Departments of Obstetrics and Gynecology, Pediatrics, and Physiology, University of Alberta, Edmonton, Alberta, Canada; 12 Department of Community Health Sciences, University of Calgary, Calgary Alberta Canada

## Abstract

**Background:**

Preterm birth is the leading cause of perinatal morbidity and mortality. Risk factors for preterm birth include a personal or familial history of preterm delivery, ethnicity and low socioeconomic status yet the ability to predict preterm delivery before the onset of preterm labour evades clinical practice. Evidence suggests that genetics may play a role in the multi-factorial pathophysiology of preterm birth. The All Our Babies Study is an on-going community based longitudinal cohort study that was designed to establish a cohort of women to investigate how a women's genetics and environment contribute to the pathophysiology of preterm birth. Specifically this study will examine the predictive potential of maternal leukocytes for predicting preterm birth in non-labouring women through the examination of gene expression profiles and gene-environment interactions.

**Methods/Design:**

Collaborations have been established between clinical lab services, the provincial health service provider and researchers to create an interdisciplinary study design for the All Our Babies Study. A birth cohort of 2000 women has been established to address this research question. Women provide informed consent for blood sample collection, linkage to medical records and complete questionnaires related to prenatal health, service utilization, social support, emotional and physical health, demographics, and breast and infant feeding. Maternal blood samples are collected in PAXgene™ RNA tubes between 18-22 and 28-32 weeks gestation for transcriptomic analyses.

**Discussion:**

The All Our Babies Study is an example of how investment in clinical-academic-community partnerships can improve research efficiency and accelerate the recruitment and data collection phases of a study. Establishing these partnerships during the study design phase and maintaining these relationships through the duration of the study provides the unique opportunity to investigate the multi-causal factors of preterm birth. The overall All Our Babies Study results can potentially lead to healthier pregnancies, mothers, infants and children.

## Background

Preterm birth remains poorly understood in modern society despite accounting for 75% of perinatal mortality [1,2]. Moreover, the rate of preterm birth, that is birth before 37 completed weeks of gestation, has been on the rise for the past 3 decades in many developed countries including Canada, the USA and European nations where current rates range between 5-12% [3]. Infants born preterm are at increased risks for neonatal complications and associated long-term morbidity [4] such as developmental delays, hearing and vision impairments, respiratory distress syndrome, cerebral palsy and retinopathy of prematurity, prolonging the financial, emotional and stress-related costs of prematurity well beyond the care received within neonatal intensive care units. The risk for adverse neonatal outcomes is inversely related to gestational age at birth with the highest morbidity among those infants delivered prior to 28 completed weeks of pregnancy (early preterm) [5] despite technological advances and improved medical treatments having greatly increased the survival of these infants [6,7]. The rate of late preterm births (34-36 weeks gestation) has also continued to climb and accounts for 75% of all preterm infants [8]. Recent studies have shown that these late preterm infants are at higher risk for adverse acute and long-term outcomes when compared to term infants [9].

The pathophysiology of preterm birth is complex due to multi-factorial causes and its heterogeneous nature. Preterm birth is often categorized as (1) iatrogenic when delivery is a consequence of medical intervention (30-35%); (2) spontaneous when it occurs after spontaneous labour with intact membranes (40-45%); or (3) the result of preterm premature rupture of the membranes (PPROM) (25-30%) [3]. While gestational diabetes, preeclampsia and intrauterine growth restriction have all been associated with iatrogenic preterm birth, the underlying causes of spontaneous preterm birth are less clear. Reported risk factors are widespread and diverse, supporting the hypothesis of gene-gene and gene-environment interactions in the pathophysiology of spontaneous preterm birth. Highly associated risk factors include a previous preterm birth or a relative with preterm birth [10] or a black racial background [11], suggesting genetic causes. Other demographic factors such as low socioeconomic status [12] and maternal stress [13] have shown an association, indicating that the environment contributes to the etiology of spontaneous preterm birth.

Evidence for a causal role of infection and inflammation in preterm birth has been reviewed in detail [14]. Both interleukin (IL)-1β and tumor necrosis factor (TNF)α are cytokines produced by the intrauterine tissues in response to bacterial infections [15,16] which stimulate prostaglandin synthesis [17] and increase uterine contractility. Several groups have investigated the relationship between a single nucleotide polymorphism (SNP) located in the promoter of the TNFα gene (G-308>A) (referred to as the TNF-2 allele) and preterm birth, but inconsistent results have been reported [18-21]. A recent meta-analysis of seven studies [22] found no association between preterm parturition and the TNF-2 allele. Similarly, studies examining polymorphisms in IL-1β and IL-6 as well as other variants have also produced inconsistent results [19,23,24].

These inconsistencies may be the result of gene-environment interactions impacting the effects of potential genetic predispositions to preterm birth. For example, Macones *et al*. reported an increased risk of preterm delivery being associated with both the TNF-2 allele and the presence of bacterial vaginosis, but not with either factor alone [25] suggesting a synergistic interaction. These findings highlight the complexity of understanding preterm birth and the need for an interdisciplinary approach, a strategy embraced by the Preterm Birth International Collaborative (PREBIC) in recently published guidelines for preterm birth research [26].

Prediction of risk for preterm birth is critical to successful clinical management. Understanding risk and protective factors is best accomplished through the development of a pregnancy cohort where asymptomatic women can be prospectively recruited and followed over time. Despite our growing awareness that infection, inflammation, stress and other risk factors contribute to the pathophysiology of preterm birth, predicting which women will deliver preterm remains an ongoing challenge. Many biomarkers for preterm birth have been suggested and explored such as fetal fibronectin [27,28], maternal corticotropin releasing hormone (CRH) [29], and cervical length [30] however all have had limitations such as low positive predictive values, low sensitivity or poor specificity [31] in addition to high inter-individual variability, which limit their translation into clinical settings. Bocking *et al*. have shown that white blood cell (leukocyte) counts [32] or the presence of infection [33] at 22-27 weeks gestation is an accurate predictor of preterm birth before 28 weeks gestation. These researchers have also shown CRH or combination of CRH and maternal age to be predictive for mid to late preterm deliveries respectively. Recent multiple-marker tests for risk of preterm birth show promise but continue to have limited predictive power for non-labouring women [34].

A clinical screen for risk of preterm delivery before women have signs or symptoms of preterm labour is needed. The evidence also suggests that an interdisciplinary approach is required to understand preterm birth pathophysiology. The purpose of this paper is to describe the methodology for community based recruitment of a pregnancy cohort, The All Our Babies Cohort Study, to examine genetic, environmental and pathophysiologic contributions to preterm birth.

## Methods/Design

### Objectives

The aims of the All Our Babies Cohort Study are

a) To investigate genetic components of preterm birth through comparisons of transcriptomic patterns at 18-22 weeks and 28-32 weeks of pregnancy between spontaneous preterm birth cases and term delivering controls,

b) To examine the association between environmental factors and preterm birth, including access to routine prenatal care and health services, maternal mental and psychosocial health during pregnancy, and;

c) To examine the relative impact of genes and environment on the risk of preterm birth by exploring the interactions between medical history, gene expression patterns and environmental variables.

### Study Design

The All Our Babies Cohort Study is a community-based prospective pregnancy cohort study to determine the environmental and genetic risks for preterm birth.

### Study Population

The study population is drawn from all women who receive prenatal viral serology testing in Calgary, Alberta, Canada. Participants reside within the Calgary city limits and surrounding rural communities. Women are recruited through a partnership with the clinical laboratory service in Calgary.

### Inclusion Criteria

Inclusion criteria for the All Our Babies Cohort Study are designed to ensure women most at risk of preterm birth are captured in the cohort.

1. Women <17 weeks 6 days gestation at time of recruitment

2. Women receiving prenatal care in Calgary

3. Women able to understand written and spoken English

4. Age 18 years or older at time of enrollment

5. One of the following pregnancy histories:

a. Nulliparous or primiparous OR

b. A personal or familial history of preterm birth

6. Singleton pregnancy

### Exclusion Criteria

1. Planned to move outside of the greater Calgary area during their pregnancy

2. Known to be carrying multiples at the time of enrollment

3. Had any of the following pre-existing medical conditions

a. Type I or Type II diabetes

b. High blood pressure or hypertension

c. Autoimmune/immune disorders: lupus, rheumatoid arthritis, Sjogren's syndrome

d. Kidney disease, chronic renal disease, nephritis, nephropathy, dialysis

e. A heart problem that was repaired by surgery

f. Chronic infection: hepatitis, HIV

### Recruitment Strategies

Prenatal clinical practice guidelines prompt evaluation of viral serology by public health laboratories. The sole provider of phlebotomy for this service in the Calgary region is Calgary Laboratory Service. Women with laboratory orders for prenatal viral serology tests are contacted by telephone by the clinical laboratory to ask for permission to release their contact information to the research staff. Women that consent to the release of their information are subsequently contacted by telephone to determine eligibility, inform them about the study and invite them to participate. Women who provide verbal consent to participate are enrolled in the study and are mailed a record of the consent form for their records. Written consent for blood collections and associated genetic analyses is obtained at the time of the first blood collection and prior to the blood draw.

### Questionnaire Data Collection

Questionnaires were developed to address the objectives of the All Our Babies Cohort Study and included input from academics, stakeholders and decision makers. These questionnaires (available on request) assess demographics, pregnancy history, service utilization, nutrition and exercise practices, mental health, social support, lifestyle and life history, and breastfeeding experiences through questions designed for this study as well as the following validated instruments: Edinburgh Postnatal Depression Scale [35], Spielberger State Anxiety Scale [36], MOS Social Support Scale [37], Perceived Stress Scale [38], T-ACE Screen for alcohol consumption risk [39], Parenting Morale Index [40], the Parental Expectations Scale for parenting self-efficacy [41], and the MCH Feeding Scale (personal communication, M. Ramsay, Feeding Problem Questionnaire 2003). The questionnaires were pilot tested prior to study commencement and revised for unclear wording. The questionnaires were designed using the Cardiff Teleform software suite (Cardiff Teleform, Version 10.1, 2007) which enables the conversion to electronic data upon completion of paper-based copies.

The first questionnaire is mailed out at study enrollment for completion before 24 weeks of gestation. Women are given reminder calls beginning at 20 weeks gestation or 3 weeks after the questionnaire is mailed, whichever is later, to answer participants' questions and encourage completion and return of the questionnaire.

The second questionnaire is mailed to all participants at 32 weeks gestation, to be completed between 34-36 weeks gestation. At 36 weeks gestation, women are contacted by telephone to provide a reminder about the questionnaire. A modified version of this questionnaire is used for women who delivered prior to receiving or completing the questionnaire.

The third questionnaire is designed for completion at four months post-partum. Women are contacted two weeks after their expected due date to determine the birthdate of their infant(s). The questionnaire is mailed to participants at 3.5 months postpartum.

### Obstetrical and Birth Record Data

The Alberta Perinatal Health (APH) database is an Alberta-based database that contains prenatal maternal and birth outcome information from all recorded births in the province. Study participants provide informed consent to access medical records. This enables the questionnaire data to be linked to their APH administrative data about birth outcomes, and to examine medical records to verify circumstances associated with preterm birth.

Due to the expected 18 month delay to populate and validate the APH database, study team members perform manual chart extractions for all preterm deliveries in the All Our Babies Cohort Study. This accelerates the collection of key medical record variables and is required to phenotype each preterm birth and enable case selection for the genetic analyses.

### Maternal Blood Specimens

Maternal blood samples are collected at 18-22 weeks gestation and at 28-32 weeks gestation either at a laboratory location or by a mobile phlebotomist. Participants are contacted at 17 and 27 weeks gestation by telephone and/or email to begin scheduling their first and second blood collections respectively. Collections can occur at any time of day and fasting is not required.

Four PAXgene™ blood RNA tubes (PreAnalytix/BD Canada, Mississauga, Ontario, Canada) are used at each blood collection. These tubes are specially designed to preserve RNA in whole blood at the time of collection. Blood is also collected into a heparin tube to isolate plasma (first collection), a serum collection tube (second collection) and into an EDTA tube for maternal DNA extraction.

### Cord Blood Specimens

Umbilical cord blood (3-5 ml) is routinely collected following hospital births to establish infant red blood cell antigens. Study participants provide informed consent to allow the researchers to access the unused portion of these blood specimens that would otherwise be discarded. These samples are stored at -80°C as a source of fetal DNA.

### Summary of Frequency and Duration of Follow-Up

All participants are asked to complete three questionnaires: at <24 weeks gestation, at 34-36 weeks gestation and at 4 months post-partum for the All Our Babies Cohort Study. Blood collections are completed between 18-22 weeks of pregnancy and between 28-32 weeks of pregnancy. Women are contacted at approximately two weeks after their estimated due date to determine the delivery date. It is anticipated that participants of the All Our Babies Cohort Study will be followed-up every two to three years to provide the opportunity for life course research on maternal, child and family outcomes.

### Methods of Protecting Against Sources of Bias

All women who receive prenatal viral serology phlebotomy by Calgary Laboratory Service and consent to be contacted by researchers are contacted via telephone. Should they meet the inclusion criteria, the women are invited to participate in the All Our Babies Cohort Study. This recruitment strategy minimizes selection bias due to self-referral or patient populations at specific clinical practices and enables a citywide and surrounding area sampling approach.

Questionnaire data is collected prospectively, eliminating the influence of birth outcome on responses and potential for differential recall bias. However, the repeat use of standardized scales may introduce a form of bias where women remember answering the questions before and past answers influence responses on subsequent questionnaires. We anticipate that misclassification errors in exposure data will not be an issue in the information collected and generated from this cohort due to well-spaced and timely use of questionnaires.

Blood collections are offered at both a permanent laboratory location and by mobile phlebotomists to ensure that women are able to provide the blood sample at a location and time that is comfortable and convenient. The mobile phlebotomist option enables evening and weekend blood collections so that work hours are not a constraint. The flexibility of the blood collection options minimizes participant loss due to inconvenience or personal schedule demands.

### Proposed Outcome Measures

a) Transcriptomic profiles from maternal blood samples collected at 18-22 weeks and 28-32 weeks of pregnancy will be compared between two groups (women who deliver at term and women who deliver preterm) for patterns predictive of preterm birth.

b) Health service utilization, maternal social support, psychosocial well-being, mental health, breastfeeding and parenting support at four months postpartum will be compared between term and preterm deliveries.

c) The relative contribution of the environmental risk and the genetic risk will be compared between term and preterm deliveries.

### Sample Size

Sample size calculations for the All Our Babies Cohort Study required complex considerations as maternal blood samples are destined for microarray analyses. Several power calculations for microarray methodologies exist, consequently sample size was determined and compared with two power calculation approaches. The first approach uses a two-class comparison model with a significance level of 0.001, a power of 0.95, one technical replicate per sample and estimated values of τ^2 ^+ 2 γ^2 ^= 0.25 and τ^2^/γ^2 ^= 4 (where τ^2 ^is the biological variance within class of log ratios and γ^2 ^is the technical variance of log ratios); the minimum sample size to identify a two-fold change in gene expression between classes is thus 25 patients in each study group [42,43]. An alternate approach is to consider the sample size required to detect a two-fold change in the expression levels of the 90% and 75% least-variable genes for a given set of false positive rates and power [42]; using previously published human tissue microarray data [43] and setting the false positive rates at 0.001 and a power of 0.9, this approach gave a minimum of 18 and 13 patients in each group to detect a two-fold change in expression levels for the 90% and 75% least-variable genes, respectively. Microarrays will be performed on RNA samples collected from 80 cases of spontaneous preterm birth and 80 matched controls (160 biological replicates) from samples collected at two time points (18-22 weeks and 28-32 weeks): a total of 320 microarrays. This sample size will allow prediction of:

1) idiopathic PTB (n~40), including

a) idiopathic PTB prior to 32 weeks gestation (n~13); and

b) idiopathic PTB between 32 and 37 weeks gestation (n~27);

2) PPROM and PTB (n~40), including

a) PPROM and PTB prior to 32 weeks gestation (n~13);

b) PPROM and PTB between 32 and 37 weeks gestation (n~27);

3) spontaneous PTB (n~80), including

a) spontaneous PTB prior to 32 weeks (n~25); and

b) spontaneous PTB between 32 and 37 weeks gestation (n~55).

Therefore, with a preterm birth rate of 9.1% in Alberta, an anticipated miscarriage rate of 5% and an anticipated loss to follow-up rate of 15% based on experience with other Alberta based cohorts [44], 2200 women will be enrolled in the All Our Babies Cohort Study. This should ensure 1800 women complete the study, of which approximately 180 will have preterm birth outcomes and approximately 120 of these will be spontaneous with or without PPROM.

The impact of access to routine prenatal care and health services on maternal variables at four months postpartum will be compared among the entire cohort. Although the sample size calculations for the All Our Babies Cohort Study were derived for the microarray analysis, the sample size of 1800 women is adequate to describe differences between groups with respect to health service utilization, maternal social support, psychosocial well-being, mental health, breastfeeding and parenting support using logistic regression [45-47].

### Planned Recruitment Rate

There are approximately 18,500 live births per year in Calgary [48]. Recruitment for the All Our Babies Cohort Study began September 2009 and is targeted for completion late 2010. The recruitment rate has averaged 36 participants/week through the community laboratory services and represents approximately 10% of births in Calgary during this period.

### Study Compliance

Strategies used to maintain participant involvement include reminder phone calls for outstanding questionnaires and providing incentives including public library gift certificates and grocery gift certificates for completed questionnaires. Mailing the questionnaires and providing postage paid envelopes has minimized time and costs to participants.

The All Our Babies Cohort Study participants are provided with two options for blood collections. Women may schedule an appointment through the research team at a central community laboratory clinic within Calgary. For participants unable or unwilling to travel to the laboratory clinic, the research team will send a certified mobile phlebotomist to the women's home or other neutral location of her choice for the blood collections. In appreciation for their time and commitment to the study, women are provided with department store gift certificates at the time of their blood collection.

Umbilical cord blood samples collected at the time of birth at local hospital births are sent to a single transfusion medicine laboratory location. The research team is notified of delivery and obtains these specimens after all clinical testing is completed. Women and their health care providers therefore do not need to do anything at the time of delivery for the study. Study team members need not be present at delivery units.

### Anticipated Rate of Loss To Follow-Up

The All Our Babies Cohort Study anticipates retaining 85% of women after completion of the first questionnaire and excluding miscarriages. Based on women's consent to participate in follow-up studies of 97% and with dedicated retention strategies, it is anticipated that no more than 10% of study participants will be lost to follow-up into the preschool years.

### Proposed Type and Frequency of Analysis

#### Transcriptomic Analysis

RNA will be extracted from samples collected from identified cases (spontaneous preterm births with or without PPROM) and controls (term deliveries). Cases and controls will be 'loosely matched' such that each group has a similar distribution of maternal ages, prepregnancy body mass indexes and smoking status. Extracted RNA will be aliquoted into three tubes - one for Agilent RNA quality assessment, one for the transcriptomic microarray, and one for validation of microarray findings by real time polymerase chain reaction. Samples will be run on Affymetrix microarrays in two batches of 40 cases and 40 controls. All statistical analyses will be conducted on the full dataset generated from the microarrays.

#### Statistical Analysis

Transcriptomic data, medical record data and self-report questionnaire data will first be analyzed univariately. Participant characteristics will be assessed and compared to the pregnant and parenting population in the province of Alberta and nationwide across Canada. Should transcriptomic signatures predictive of preterm delivery phenotypes be elucidated from the microarray analyses and validation, gene-environment interactions will be explored by integrating the transcriptomic data with the questionnaire and medical record datasets using appropriate statistical analytical techniques.

### Potential Risks To The Safety of Particiants Involved In The Study

#### Medical Risks

Participation in the All Our Babies Cohort Study does not alter women's medical care during pregnancy. Participants are informed about the routine risks during phlebotomy (eg. risk of bruising or infection). There are also no direct medical benefits to the participants nor their infants for participating in the All Our Babies Cohort Study.

#### Confidentiality

A coded study ID is assigned to each participant at the time of enrollment. This ID is used to identify all questionnaire data, biological specimens and extracted information from participants' medical records. The study ID key is contained in a password protected file on password protected computers in a secure office area. All questionnaires and biological specimens are stored in locked cabinets/freezers in secure areas. Only study team members have access to the identifying data collected in this study.

Information from participants' medical records is obtained through linkage with the APH database. At the study's end, Alberta Health and Wellness will extract the requested information from their database for our study participants. Published results will report only aggregate information and will not identify individual participants. Participants will be notified of aggregated study findings through the distribution of a study summary.

### Significance of The Study

The rate of preterm birth is on the rise in the developed world. Alberta has the highest provincial preterm birth rate in Canada, the causes of which remain unexplained. The All Our Babies Cohort Study is strategically situated to examine the environmental, medical and genetic factors impacting preterm birth in Alberta and seeks to exploit their predictive properties for future clinical screening of all pregnant women. Should gene expression patterns predictive of spontaneous preterm birth be elucidated or gene-environment interactions protective against or synergistic for a spontaneous preterm birth outcome be discovered, the detailed phenotypic information will inform validation endeavors. This could potentially expedite the journey from basic science discovery to clinical utility, a translation process which to date has had limited success in the applications of 'omics technologies in maternal-fetal newborn health. In addition, this study will improve our understanding of how social support and social services impact spontaneous preterm birth in Calgary.

The delivery of health services in the province of Alberta is currently undergoing reorganization making the assessment of women's needs during pregnancy very timely to inform service and program development and change of healthcare policies. The comprehensive questionnaire data obtained from participants in the All Our Babies Cohort Study combined with data from medical records provides a concise record of local health practices and the impact of practice on maternal-fetal and newborn health. The results of the study will provide the opportunity to describe the mental health and psychosocial characteristics of mothers living in Calgary as well as estimates of the impact of these characteristics on birth and the parenting experience. The All Our Babies Cohort Study will also record women's prenatal care experience in Calgary and identify barriers and facilitators to accessing prenatal care. In addition, the information will enable evaluation of the relationships between access to routine prenatal care on postpartum depression, parenting morale, anxiety, stress, social support, lifestyle choices such as smoking, drug use and alcohol consumption, breastfeeding initiation, duration and challenges and postpartum use of health care services. Furthermore the research team is uniquely positioned to report directly to Alberta Health Services stakeholders, ensuring knowledge gained from this research is used to inform programs and practice.

### Ethics

This study was approved by the following ethics review boards: Health Research Ethics Board (University of Alberta, Edmonton, Canada; Ethics ID 7515), Conjoint Health Research Ethics Board (University of Calgary, Calgary; Ethics ID 22128), Health Records Services (Calgary; Ethics ID 2265), Child Health Research Office (Calgary; Ethics ID #E-22128), and the Calgary Laboratory Services Ethics and Privacy Office (Calgary).

Participants are asked to provide verbal consent to participate in the study over the phone at the time of recruitment, and completion and return of the questionnaires signifies implied consent. At the time of the first blood collection, participants are required to give signed consent to the blood collections, access to medical records and corresponding analyses. Participants are given copies of the consent forms to which they provided informed consent over the phone and in writing.

### Study Timeline

The All Our Babies Cohort Study began in September 2009. Participants are involved in the study for approximately one year's time. It is anticipated that sample collections will be completed by spring 2011, all participants will have delivered their infant(s) by summer 2011 and data collection will be completed by fall 2011. Participants have been asked about their interest in participating in follow-up studies, potentially allowing for further research on early determinants of child and adult health outcomes.

## Discussion

Complex preterm phenotypes are determined by genetic, physiological and environmental factors interacting at the cellular, organ, systemic, and lifestyle levels. The All Our Babies Cohort Study is gathering extensive detailed data on the gene expression profiles, medical history, lifestyle, demographics, physical and mental well-being, and neighbourhoods of the participants to predict preterm birth in asymptomatic women. The integration of these diverse data types will create an improved understanding of both maternal well-being in the perinatal period and of the cascade of the pathophysiology of preterm birth, the ultimate goal being improved maternal-fetal-newborn health outcomes.

The All Our Babies Cohort Study is the optimal study design to facilitate the integration of biological, medical and social data. The research team, community laboratory service and provincial health service provider are intimately partnered to facilitate multiple aspects of the study process. Recruitment, biological sample collection, and accessing medical record data are made possible by the academic-clinical collaborations that are the foundation of the All Our Babies Cohort Study. The implementation of this study protocol represents a successful example of how academic-clinical partnerships can increase efficiency in the research process, expediting the generation of study findings by streamlining the recruitment and collection phases of the study. Monthly meetings, routine newsletters, local and provincial committee membership and established relationships with key stakeholders, including program designers and decision makers, increase the likelihood of rapid translation of research findings into policy and programs.

## Competing interests

Financial competing interests: In accordance with the conditions of the funding agency (Alberta Innovates - Health Solutions), the research will be evaluated by University Technologies Inc, Calgary, AB, and Technology Edmonton, AB, for possible protection as intellectual property.

## Authors' contributions

SCT is responsible for the overall integrity, progress and timely completion of the study. SCT, CEP, ADB, SJL, SMD, AWL, and DMO are involved in the study design, the acquisition of funding, providing advice on methodological issues, and contributing to the interpretation of study results. SCT, KMH, HLK, DAM and JES were involved in the design, content and piloting of the questionnaires. AWL is responsible for all lab-based queries. SWM will be responsible for the management, coding, linkage and analysis of data. SKG was involved in the study design, is responsible for the daily management of the study, ensuring protocol compliance, high-quality and complete data collection, and will undertake analysis of data. Study investigators are required to participate in meetings related to study issues and progress as needed. All authors have read and approved the final manuscript.

## Pre-publication history

The pre-publication history for this paper can be accessed here:

http://www.biomedcentral.com/1471-2393/10/87/prepub

## References

[B1] Goldenberg RL The management of preterm labor Obstet Gynecol 2002 100 5 Pt 1 1020 1037 10.1016/S0029-7844(02)02212-3 12423870

[B2] McCormick MC The contribution of low birth weight to infant mortality and childhood morbidity N Engl J Med 1985 312 2 82 90 10.1056/NEJM198501103120204 3880598

[B3] Goldenberg RL Culhane JF Iams JD Romero R Epidemiology and causes of preterm birth Lancet 2008 371 9606 75 84 10.1016/S0140-6736(08)60074-4 18177778 PMC7134569

[B4] Ward RM Beachy JC Neonatal complications following preterm birth BJOG 2003 110 Suppl 20 8 16 12763105 10.1016/s1470-0328(03)00012-0

[B5] Stoll BJ Hansen NI Bell EF Shankaran S Laptook AR Walsh MC Hale EC Newman NS Schibler K Carlo WA Neonatal outcomes of extremely preterm infants from the NICHD Neonatal Research Network Pediatrics 126 3 443 456 10.1542/peds.2009-2959 20732945 PMC2982806

[B6] Muglia LJ Katz M The enigma of spontaneous preterm birth N Engl J Med 362 6 529 535 10.1056/NEJMra0904308 20147718

[B7] Iams JD Romero R Culhane JF Goldenberg RL Primary, secondary, and tertiary interventions to reduce the morbidity and mortality of preterm birth Lancet 2008 371 9607 164 175 10.1016/S0140-6736(08)60108-7 18191687

[B8] Davidoff MJ Dias T Damus K Russell R Bettegowda VR Dolan S Schwarz RH Green NS Petrini J Changes in the gestational age distribution among U.S. singleton births: impact on rates of late preterm birth, 1992 to 2002 Semin Perinatol 2006 30 1 8 15 10.1053/j.semperi.2006.01.009 16549207

[B9] Bastek JA Sammel MD Pare E Srinivas SK Posencheg MA Elovitz MA Adverse neonatal outcomes: examining the risks between preterm, late preterm, and term infants Am J Obstet Gynecol 2008 199 4 367 e361-368 10.1016/j.ajog.2008.08.002 18928976

[B10] Goldenberg RL Rouse DJ Prevention of premature birth N Engl J Med 1998 339 5 313 320 10.1056/NEJM199807303390506 9682045

[B11] Demissie K Rhoads GG Ananth CV Alexander GR Kramer MS Kogan MD Joseph KS Trends in preterm birth and neonatal mortality among blacks and whites in the United States from 1989 to 1997 Am J Epidemiol 2001 154 4 307 315 10.1093/aje/154.4.307 11495853

[B12] Kramer MS Goulet L Lydon J Seguin L McNamara H Dassa C Platt RW Chen MF Gauthier H Genest J Socio-economic disparities in preterm birth: causal pathways and mechanisms Paediatr Perinat Epidemiol 2001 15 Suppl 2 104 123 10.1046/j.1365-3016.2001.00012.x 11520404

[B13] Copper RL Goldenberg RL Das A Elder N Swain M Norman G Ramsey R Cotroneo P Collins BA Johnson F The preterm prediction study: maternal stress is associated with spontaneous preterm birth at less than thirty-five weeks' gestation. National Institute of Child Health and Human Development Maternal-Fetal Medicine Units Network Am J Obstet Gynecol 1996 175 5 1286 1292 10.1016/S0002-9378(96)70042-X 8942502

[B14] Romero R Espinoza J Kusanovic JP Gotsch F Hassan S Erez O Chaiworapongsa T Mazor M The preterm parturition syndrome BJOG 2006 113 Suppl 3 17 42 17206962 10.1111/j.1471-0528.2006.01120.x PMC7062298

[B15] Romero R Wu YK Brody DT Oyarzun E Duff GW Durum SK Human decidua: a source of interleukin-1 Obstet Gynecol 1989 73 1 31 34 2642326

[B16] Casey ML Cox SM Beutler B Milewich L MacDonald PC Cachectin/tumor necrosis factor-alpha formation in human decidua. Potential role of cytokines in infection-induced preterm labor J Clin Invest 1989 83 2 430 436 10.1172/JCI113901 2913048 PMC303698

[B17] Hansen WR Keelan JA Skinner SJ Mitchell MD Key enzymes of prostaglandin biosynthesis and metabolism. Coordinate regulation of expression by cytokines in gestational tissues: a review Prostaglandins Other Lipid Mediat 1999 57 4 243 257 10.1016/S0090-6980(99)00008-8 10402218

[B18] Engel SA Erichsen HC Savitz DA Thorp J Chanock SJ Olshan AF Risk of spontaneous preterm birth is associated with common proinflammatory cytokine polymorphisms Epidemiology 2005 16 4 469 477 10.1097/01.ede.0000164539.09250.31 15951664

[B19] Menon R Velez DR Simhan H Ryckman K Jiang L Thorsen P Vogel I Jacobsson B Merialdi M Williams SM Multilocus interactions at maternal tumor necrosis factor-alpha, tumor necrosis factor receptors, interleukin-6 and interleukin-6 receptor genes predict spontaneous preterm labor in European-American women Am J Obstet Gynecol 2006 194 6 1616 1624 10.1016/j.ajog.2006.03.059 16731080

[B20] Menon R Velez DR Morgan N Lombardi SJ Fortunato SJ Williams SM Genetic regulation of amniotic fluid TNF-alpha and soluble TNF receptor concentrations affected by race and preterm birth Hum Genet 2008 124 3 243 253 10.1007/s00439-008-0547-z 18807256

[B21] Dizon-Townson DS Major H Varner M Ward K A promoter mutation that increases transcription of the tumor necrosis factor-alpha gene is not associated with preterm delivery Am J Obstet Gynecol 1997 177 4 810 813 10.1016/S0002-9378(97)70273-4 9369824

[B22] Menon R Merialdi M Betran AP Dolan S Jiang L Fortunato SJ Williams S Analysis of association between maternal tumor necrosis factor-alpha promoter polymorphism (-308), tumor necrosis factor concentration, and preterm birth Am J Obstet Gynecol 2006 195 5 1240 1248 10.1016/j.ajog.2006.05.037 17074545

[B23] Jamie WE Edwards RK Ferguson RJ Duff P The interleukin-6--174 single nucleotide polymorphism: cervical protein production and the risk of preterm delivery Am J Obstet Gynecol 2005 192 4 1023 1027 10.1016/j.ajog.2005.01.035 15846175

[B24] Dolan SM Hollegaard MV Merialdi M Betran AP Allen T Abelow C Nace J Lin BK Khoury MJ Ioannidis JP Synopsis of Preterm Birth Genetic Association Studies: The Preterm Birth Genetics Knowledge Base (PTBGene) Public Health Genomics 2010 13 7-8 514 523 10.1159/000294202 20484876

[B25] Macones GA Parry S Elkousy M Clothier B Ural SH Strauss JF A polymorphism in the promoter region of TNF and bacterial vaginosis: preliminary evidence of gene-environment interaction in the etiology of spontaneous preterm birth Am J Obstet Gynecol 2004 190 6 1504 1508 discussion 1503A 10.1016/j.ajog.2004.01.001 15284722

[B26] Pennell CE Jacobsson B Williams SM Buus RM Muglia LJ Dolan SM Morken NH Ozcelik H Lye SJ Relton C Genetic epidemiologic studies of preterm birth: guidelines for research Am J Obstet Gynecol 2007 196 2 107 118 10.1016/j.ajog.2006.03.109 17306646

[B27] Kiefer DG Vintzileos AM The utility of fetal fibronectin in the prediction and prevention of spontaneous preterm birth Rev Obstet Gynecol 2008 1 3 106 112 19015761 PMC2582650

[B28] Honest H Bachmann LM Gupta JK Kleijnen J Khan KS Accuracy of cervicovaginal fetal fibronectin test in predicting risk of spontaneous preterm birth: systematic review BMJ 2002 325 7359 301 10.1136/bmj.325.7359.301 12169504 PMC117763

[B29] Hobel CJ Dunkel-Schetter C Roesch SC Castro LC Arora CP Maternal plasma corticotropin-releasing hormone associated with stress at 20 weeks' gestation in pregnancies ending in preterm delivery Am J Obstet Gynecol 1999 180 1 Pt 3 S257 263 10.1016/S0002-9378(99)70712-X 9914629

[B30] Durnwald CP Walker H Lundy JC Iams JD Rates of recurrent preterm birth by obstetrical history and cervical length Am J Obstet Gynecol 2005 193 3 Pt 2 1170 1174 10.1016/j.ajog.2005.06.085 16157132

[B31] Herbst A Nilsson C Diagnosis of early preterm labour BJOG 2006 113 Suppl 3 60 67 17206967 10.1111/j.1471-0528.2006.01125.x

[B32] Hill JL Campbell MK Zou GY Challis JR Reid G Chisaka H Bocking AD Prediction of preterm birth in symptomatic women using decision tree modeling for biomarkers Am J Obstet Gynecol 2008 198 4 468 e461-467; discussion 468 e467-469 10.1016/j.ajog.2008.01.007 18395044

[B33] Campbell MK Challis JR DaSilva O Bocking AD A cohort study found that white blood cell count and endocrine markers predicted preterm birth in symptomatic women J Clin Epidemiol 2005 58 3 304 310 10.1016/j.jclinepi.2004.06.015 15718120

[B34] Goldenberg RL Iams JD Mercer BM Meis PJ Moawad A Das A Miodovnik M Vandorsten PJ Caritis SN Thurnau G The Preterm Prediction Study: toward a multiple-marker test for spontaneous preterm birth Am J Obstet Gynecol 2001 185 3 643 651 10.1067/mob.2001.116752 11568793

[B35] Cox JL Holden JM Sagovsky R Detection of postnatal depression. Development of the 10-item Edinburgh Postnatal Depression Scale Br J Psychiatry 1987 150 782 786 10.1192/bjp.150.6.782 3651732

[B36] Spielberger CG Lushene R State-trait anxiety inventory for adults (Form X) 1970 Palo Alto, CA

[B37] Sherbourne CD Stewart AL The MOS social support survey Soc Sci Med 1991 32 6 705 714 10.1016/0277-9536(91)90150-B 2035047

[B38] Cohen S Kamarck T Mermelstein R A global measure of perceived stress J Health Soc Behav 1983 24 4 385 396 10.2307/2136404 6668417

[B39] Sokol RJ Martier SS Ager JW The T-ACE questions: practical prenatal detection of risk-drinking Am J Obstet Gynecol 1989 160 4 863 868 discussion 868-870 2712118 10.1016/0002-9378(89)90302-5

[B40] Trute B Hiebert-Murphy D Predicting family adjustment and parenting stress in childhood disability services using brief assessment tools Journal of Intellectual and Developmental Disability 2005 30 217 225 10.1080/13668250500349441

[B41] Reece SM The parent expectations survey: a measure of perceived self-efficacy Clin Nurs Res 1992 1 4 336 346 10.1177/105477389200100404 1483137

[B42] Dobbin K Simon R Sample size determination in microarray experiments for class comparison and prognostic classification Biostatistics 2005 6 1 27 38 10.1093/biostatistics/kxh015 15618525

[B43] Wei C Li J Bumgarner RE Sample size for detecting differentially expressed genes in microarray experiments BMC Genomics 2004 5 1 87 10.1186/1471-2164-5-87 15533245 PMC533874

[B44] Tough SC Siever JE Johnston DW Retaining women in a prenatal care randomized controlled trial in Canada: implications for program planning BMC Public Health 2007 7 148 10.1186/1471-2458-7-148 17617914 PMC1939989

[B45] Concato J Peduzzi P Holford TR Feinstein AR Importance of events per independent variable in proportional hazards analysis. I. Background, goals, and general strategy J Clin Epidemiol 1995 48 12 1495 1501 10.1016/0895-4356(95)00510-2 8543963

[B46] Peduzzi P Concato J Feinstein AR Holford TR Importance of events per independent variable in proportional hazards regression analysis. II. Accuracy and precision of regression estimates J Clin Epidemiol 1995 48 12 1503 1510 10.1016/0895-4356(95)00048-8 8543964

[B47] Peduzzi P Concato J Kemper E Holford TR Feinstein AR A simulation study of the number of events per variable in logistic regression analysis J Clin Epidemiol 1996 49 12 1373 1379 10.1016/S0895-4356(96)00236-3 8970487

[B48] Alberta Health Pregnancies and Birth Surveillance Report http://www.health.alberta.ca/documents/Reproductive-Health-2009-Update.pdf

